# Avian Metapneumovirus Subgroup C Infection in Chickens, China

**DOI:** 10.3201/eid1907.121126

**Published:** 2013-07

**Authors:** Li Wei, Shanshan Zhu, Xv Yan, Jing Wang, Chunyan Zhang, Shuhang Liu, Ruiping She, Fengjiao Hu, Rong Quan, Jue Liu

**Affiliations:** Beijing Municipal Key Laboratory for Prevention and Control of Infectious Diseases in Livestock and Poultry, Beijing, China (L. Wei, S. Zhu, X. Yan, J. Wang, C. Zhang, S. Liu, R. Quan, J. Liu);; Beijing Academy of Agriculture and Forestry Sciences, Beijing (L. Wei, S. Zhu, X. Yan, J. Wang, C. Zhang, S. Liu, R. Quan, J. Liu);; China Agricultural University, Beijing (R. She, F. Hu)

**Keywords:** Avian metapneumovirus subgroup C, Pneumovirinae, Paramyxoviridae, viruses, chickens, M gene sequencing, pathogenesis, China

## Abstract

Avian metapneumovirus causes acute respiratory tract infection and reductions in egg production in various avian species. We isolated and characterized an increasingly prevalent avian metapneumovirus subgroup C strain from meat-type commercial chickens with severe respiratory signs in China. Culling of infected flocks could lead to economic consequences.

Avian metapneumovirus (aMPV), in the subfamily *Pneumovirinae* of the family *Paramyxoviridae*, is associated with acute respiratory tract infection as well as reductions in egg production in turkeys, chickens, and ducks (*1*). aMPV contains a nonsegmented, single-stranded, negative-sense RNA genome of approximately 13 kb in length, organized as 3′-leader-N-P-M-F-M2-SH-G-L-trailer-5′ (*2*). Based on genetic and antigenic properties, aMPV can be classified into 4 subgroups: A, B, C, and D. After its first detection in South Africa in 1978 (*3*), different subgroups of aMPV, mainly A or B, were reported in Europe, Asia, and some other parts of the world in turkeys and chickens. Subgroup C aMPV was first reported in turkeys in the United States in 1996 (*4*) and subsequently isolated from farmed ducks in France (*5*) and pheasants in South Korea (*6*), as well as some wild birds (e.g., American coots, American crows, Canada geese, cattle egrets, and sparrows). These isolates are different both genetically and antigenically from subgroups A and B. Here, we report the isolation and characterization of aMPV subgroup C (aMPV-C) in chickens in China.

## The Study

During February–April 2012, severe respiratory infection in chickens was observed on some local meat-type commercial chicken farms in southeastern China. The affected chickens ranged in age from 20 to >60 days. Clinical signs were an acute and severe respiratory disease characterized by nasal and ocular discharge, foamy conjunctivitis, inflamed eyes, facial congestion, tracheal rales, swollen infraorbital sinuses, and visible yellow to white caseous discharge from the nasal sinuses or trachea. Illness rates were 30%–80% in different chicken flocks, but mortality rates were often <15%.

On a farm where yellow-feathered chickens were raised, we collected 5 nasal turbinate samples from birds that were 48 days old for further laboratory testing. These nasal turbinate samples were resuspended in minimum essential medium, and total RNA was extracted for detection of aMPV by using reverse transcription PCR (RT-PCR) subtyping as described (*7*). All 5 samples (100%) were positive for aMPV-C. In addition, the nasal turbinate samples were inoculated into Vero cell lines for 5 blind passages, and aMPV viral antigens were further detected by immunofluorescent assay by using a rabbit polyclonal antibody raised against a polypeptide located in the N protein of all aMPV subgroups (*8*). We also used PCR or RT-PCR to detect potentially related viruses (avian influenza virus subtypes H5 and H9, Newcastle disease virus, infectious laryngotracheitis virus, and infectious bronchitis virus) and used RNA or DNA isolated from homologous virus stocks as positive controls. The extracted samples did not react with the respective virus-specific primers, further indicating that aMPV-C may be a major pathogen in these affected chickens.

The full-length sequences of matrix (M) protein gene from these nasal turbinate samples were further determined and were genetically identical. The M gene sequence from the aMPV-C isolate designated as strain JC was submitted to GenBank under accession no. JX422020. Phylogenetic and molecular evolutionary analyses were conducted by using MEGA 5.10 (www.megasoftware.net). This gene has a length of 765 nucleotides (nt) encoding 254 amino acids (aa), which shared 96.0%–96.7% nt identity with aMPV-C isolates from duck, turkey, pheasant, and wild bird samples, but 70.6%–71.7% nt identity with the aMPV subgroup A and B isolates.

The chicken isolate strain JC is more closely related to human metapneumovirus (hMPV) isolates (76.6%–78.5%) than other aMPV-C isolates (75.5%–77.8%). Notably, the chicken isolate JC showed the highest identity (78.5%) to hMPV strain BJ1816, which was isolated in China. In addition, there is higher identity (98.3%–99.0%) among other aMPV-C isolates than when compared to isolate JC (96.0%–96.7%). At the amino acid level, the M protein of isolate JC shared 98.0% aa identity with the duck isolate from France, 99.2%–99.6% aa identity with the turkey and pheasant isolates, and 99.2%–99.6% aa identity with the wild bird aMPV-C isolates but 76.9%–78.4% aa identity with the aMPV subgroup A and B isolates.

**Figure 1:**
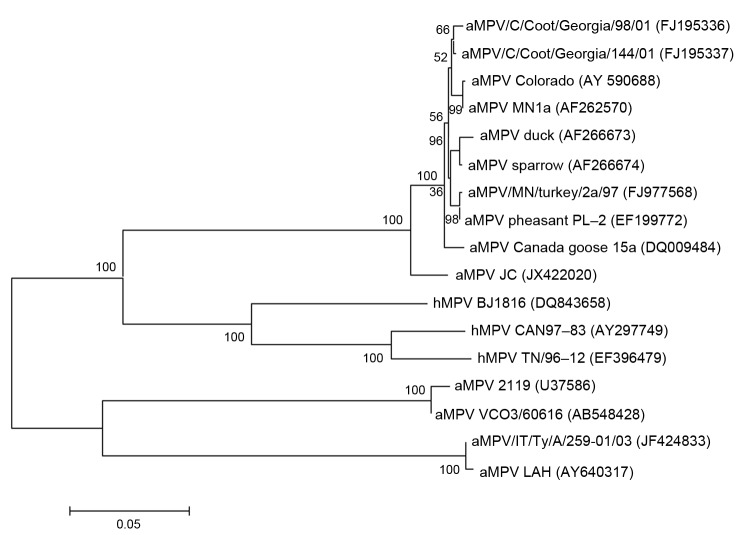
Genetic relatedness between the matrix (M) protein gene of members of avian metapneumoviruses (aMPV) and human metapneumovirus (hMPV). Phylogenetic tree was constructed on the basis of the neighbor-joining clustering method by using MEGA 5.10 software (www.megasoftware.net). Bootstrap values (based on 500 replicates) are indicated at each branching point. Reference strains obtained from GenBank are indicated. The M sequence of the isolate JC used in the phylogenetic analysis has been deposited in GenBank under accession no. JX422020. Scale bar indicates estimated phylogenetic divergence.

The phylogenetic tree of the nt sequences of aMPV representative subgroups as well as hMPV was constructed by using the neighbor-joining clustering method (Figure 1). The chicken aMPV strain JC formed 1 cluster with other aMPV-C viruses. Within the cluster, the isolate JC formed separate subclusters more similar to wild birds such as the Canada goose, suggesting that the aMPV-C might be derived from wild birds. However, the origin of the aMPV-C infection in chickens should be further studied.

We further determined the pathogenesis of the aMPV-C strain JC in specific-pathogen–free (SPF) chickens. A total of 30 two-week-old SPF chickens were randomly divided into 2 groups and placed in the animal housing units of the Institute of Animal Husbandry and Veterinary Medicine, Beijing Academy of Agriculture and Forestry Sciences. Handling of birds was in accordance with the Guidelines of Animal Care and Use Committee of the institute. One group was intranasally inoculated with the aMPV-C strain JC at a dose of 10^4.25^ by using tissue culture infectious dose 50 assay, whereas the other group was mock inoculated with 200 μL of Vero cell supernatant. Some of the inoculated chickens showed symptoms such as nasal discharge, sinus swelling, and watery eyes at 3–7 days postinoculation (dpi). Nasal discharge and oropharyngeal swabs were collected at 3, 5, and 7 dpi, and viral replication was detected by using RT-PCR. All tracheae and lung samples were collected from 5 birds each time at 3, 5, and 7 days dpi for histopathologic examination and immunohistochemical staining by using standard procedures.

**Figure 2:**
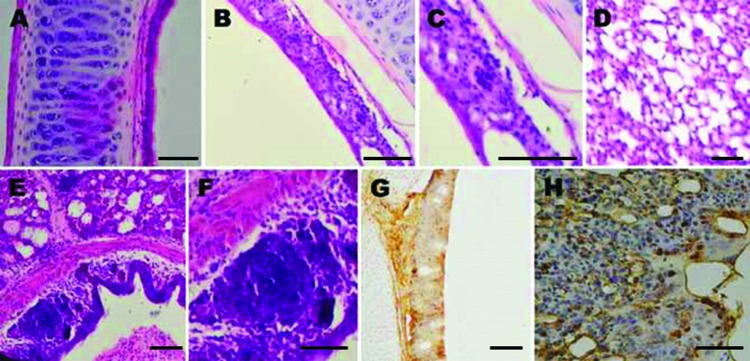
Histological appearance and immunohistochemical staining of respiratory tract samples collected from chickens before and after inoculation with avian metapneumovirus (aMPV) subgroup C, China. A) Trachea section from an uninoculated chicken shows intact ciliated epithelium. B) At 5 days’ postinoculation, loss of cilia, architectural disruption, and infiltration of inflammatory cells were seen in most of the epithelium and submucosa of inoculated chickens. C) Same lymphoid cell infiltration in trachea as in panel B, showing large numbers of lymphocytes in the epithelium. D) Lung section from an uninoculated chicken shows no significant inflammation. E) At 5 days’ postinoculation, inflammatory infiltration including lymphocytes, as well as scattered macrophages and heterophils were seen in most of the lungs of inoculated chickens. F) Same inflammatory infiltration in lung as in panel E, showing large numbers of lymphocytes in the bronchial submucosa of lung. G) Trachea tissue of aMPV-inoculated chicken shows many positive cells for aMPV antigen. H) Lung tissue of aMPV-inoculated chicken shows many positive cells for aMPV antigen. Scale bars indicate 80 μm.

Histopathological findings were characterized by mild to severe inflammation in the tracheae and lungs (Figure 2), including disruption of the epithelial architecture, sloughing of epithelial cells, loss of ciliation, and infiltration of inflammatory cells. We documented hemorrhage, infiltration of lymphoid cells, and hyperplasia in the tracheal epithelium and propria lamina of the aMPV-C–infected chickens. Examination of the lungs showed peribronchial lymphoplasmocytic infiltrates, edematous thickening of the bronchial submucosa, and lymphoid cell hyperplasia. Diffuse mild expansion of the alveolar interstitium caused by mononuclear cell infiltrates and edema was also observed. Sloughed epithelial cells, heterophils, macrophages, and amorphous debris were visible in the bronchial lumens. Immunohistochemical staining by using rabbit antibody against a polypeptide located in aMPV N protein (*8*) revealed that viral antigen was detected in both morphologically normal and degenerated respiratory epithelial cells (Figure 2). In addition, mucus cells, basal cells, and luminal cellular debris that included sloughed epithelial cells and macrophages stained positive for aMPV antigen. Research data suggested that aMPV showed no good replication ability in chickens (*9*,*10*), and that clinical signs induced by aMPV alone are not apparent in chickens. In the present study, we found that the chicken aMPV-C isolate caused severe respiratory infection and pathological inflammatory lesions in chickens, indicating that the chicken aMPV-C isolate has more severe pathogenicity than other aMPV subgroup isolates for chickens. These observations are similar to those made with hMPV infection in animal models (*11*,*12*). However, the exact mechanism needs to be further studied.

## Conclusions

We identified infection with subgroup C aMPV infection in local meat-type commercial chickens with variable severe respiratory signs in China. These findings show that aMPV-C viruses are a potential hazard for chickens, leading to ecological and economic issues. Therefore, more epidemiologic and molecular studies are needed to further assess economic losses caused by aMPV-C infections and determine the origin, distribution, and diversity of aMPV-C viruses in chickens.
